# Integrated Care Strategies for Unilateral Cleft Lip and Palate: A Multidisciplinary Treatment Approach for Profile Correction

**DOI:** 10.7759/cureus.55473

**Published:** 2024-03-04

**Authors:** Japneet K Kaiser, Ranjit H Kamble, Karthika Nambiar, Sumukh Nerurkar, Dhwani Suchak, Srushti Atole

**Affiliations:** 1 Department of Orthodontics and Dentofacial Orthopedics, Sharad Pawar Dental College and Hospital, Datta Meghe Institute of Higher Education & Research, Wardha, IND

**Keywords:** multidisciplinary treatment, adult orthodontics, alveolar cleft, class iii skeletal pattern, unilateral cleft

## Abstract

Cleft lip and palate (CLP) is a prevalent congenital craniofacial deformity that can be unilateral or bilateral. This case report highlights the interdisciplinary approach to managing a 24-year-old male with unilateral CLP (UCLP), emphasizing the complexity of associated dental and skeletal challenges. The patient had undergone multiple surgeries, including lip closure at three months, palate repair at seven years, and alveolar bone grafting at 12 years. Clinical assessments revealed a retruded maxilla, an absence of lateral incisors, and scars from previous surgeries. Radiographic evaluations indicated a Class III skeletal pattern and confirmed the presence of a cleft on the left side. Orthodontic treatment commenced with maxillary arch alignment, followed by Le Fort I surgery to address maxillary retrusion, correct skeletal malocclusion, and close the alveolar cleft space. The post-surgical phase involved orthodontic adjustments, crossbite correction, and alignment of the mandibular arch. Despite the discontinuation of treatment due to the patient’s relocation, the interdisciplinary collaboration achieved significant improvements, including a corrected facial profile, maxillary advancement, closure of the cleft space, and enhanced dental alignment. The patient’s confidence and functionality were positively impacted. This case underscores the importance of a coordinated interdisciplinary approach to addressing the multifaceted challenges associated with UCLP, aiming to optimize both aesthetic and functional outcomes for improved patient well-being.

## Introduction

Asia is more likely than other racial groups to have cleft lip and palate (CLP), the most common congenital craniofacial deformity, with occurrences of 1.33 per 1,000 live births [1]. These abnormalities can be features of several genetically defined disorders and are caused by both environmental and genetic factors. Clefts can develop between weeks four and 12 of pregnancy due to the lack of fusion between the palatal units in the secondary palate or the medial nasal and maxillary processes in the main palate (lip and premaxilla). Depending on how severe they are, clefts can be bilateral or unilateral, partial, or complete. With a frequency of 33%, unilateral CLP (UCLP) is the most common cleft, dividing the upper maxilla into greater (noncleft side) and lesser (cleft side) portions. The lack of tissues and intrinsic growth potential, as well as early reconstructive surgery, are associated with the insufficiency of maxillofacial growth in patients with UCLP [2]. Therefore, either the cleft or the reconstructive surgery may be to blame for the lack of maxillofacial growth in the cleft population. However, CLP impacts craniofacial and dentoalveolar development. As a result, cleft individuals typically experience dental anomalies such as hypodontia, malformations, and aberrant eruption patterns more commonly than the general population. Developmental problems can affect the lateral incisor bud, which grows in the dentoalveolar cleft region. The most frequent anomaly in cleft patients is a congenitally absent maxillary lateral incisor on the cleft side; supernumerary teeth in the cleft region are the second most common anomaly. Other tooth changes include those related to size (microdontia), placement (mesial or distal to the cleft), shape (pegged or conical teeth), and eruption timing. In addition to posing an aesthetic risk, these aberrations may result in restorative, functional, and periodontal issues. To address all these problems, UCLP patients need interdisciplinary care, which includes occlusal rehabilitation, i.e., correction of the occlusal plane, to meet their functional and aesthetic demands. The major objective of treatment is to achieve stability, avoid relapse, and minimize the negative effects on the patient’s social life [3]. However, the results of various treatments vary greatly because of their complexity. This clinical case study served to highlight the use of an interdisciplinary strategy in treating an adult patient who had a long list of complex problems, including UCLP.

## Case presentation

**Figure 1:**
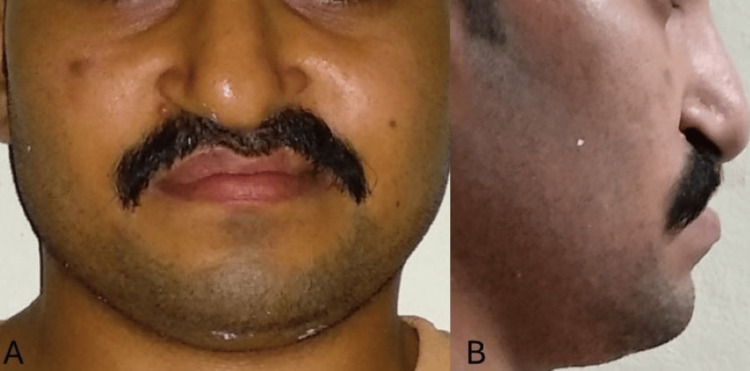
Pre-treatment extra-oral photographs (A) Frontal. (B) Profile.

A 24-year-old male presented to the Department of Orthodontics and Dentofacial Orthopedics with the chief complaint of poor aesthetics. There was no significant dental history. The patient had a medical history of surgery at the age of three months for lip closure, at seven years of age for palate repair, and at 12 years for alveolar bone grafting (ABG). There was no significant family history with similar complaints or any facial deformities. Extra-oral examination showed a straight to concave profile due to retruded maxilla, competent lips, mesocephalic head form and mesoscopic face form, acute nasolabial angle, average mentolabial sulcus, scar on lip due to lip repair surgery on the left side, no abnormality of the temporomandibular joint (TMJ) on opening or closing of the mouth, and no abnormality found on palpitation of the TMJ (Figure 1).

**Figure 2:**
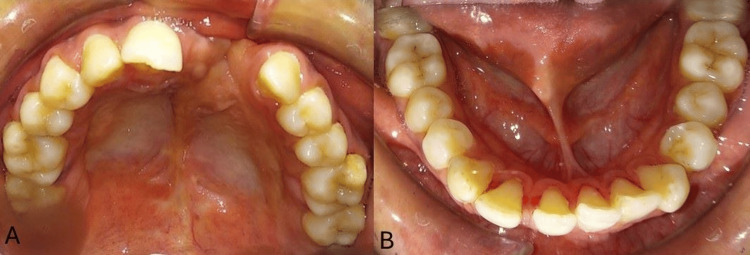
Pre-treatment intra-oral photographs (A) Maxillary arch. (B) Mandibular arch.

**Figure 3:**
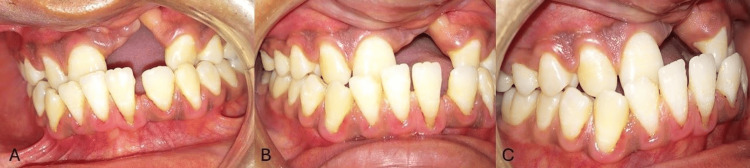
Pre-treatment intra-oral photographs (A) Right occlusal. (B) Frontal occlusal. (C) Left occlusal.

**Figure 4:**
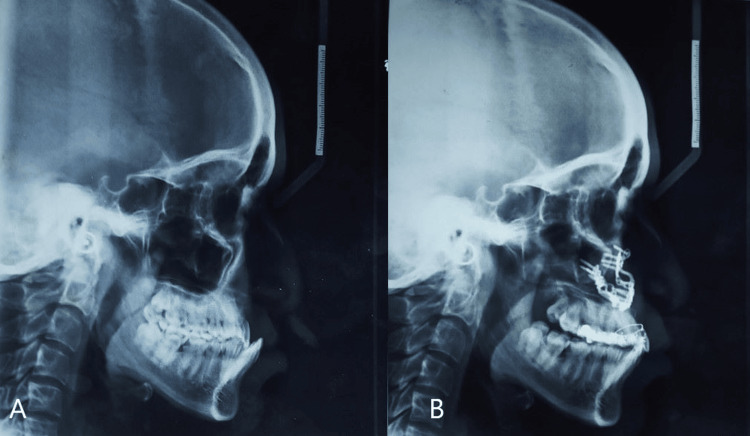
Pre- and post-treatment lateral cephalogram (A) Pre-treatment. (B) Post-treatment.

On intra-oral examination, the maxillary arch showed the absence of lateral incisors on both sides and the left central incisor and the presence of a right central incisor; both canines, both first and second premolars, were seen bilaterally, and both first and second molars were present bilaterally. The mandibular arch had all the teeth present until the second molars. The molar and canine relations were classified as Class III. No abnormality in the anatomical shape of teeth was seen. Both maxillary and mandibular arches had spaces in the anterior teeth region. The presence of a scar on the palate from palate repair surgery was seen on the left side of the upper arch, extending to the soft palate (Figure 2 and Figure 3). Furthermore, radiographs were taken, i.e., lateral cephalogram, for evaluation of skeletal problems and in relation to dental problems. The lateral cephalogram showed a retruded maxilla, dished in face profile because of reduced zygomatic prominence, proclined mandibular incisors, a tipped-down nose, increased mandibular angle, lower lip prominence, and a Class III skeletal pattern (Figure 4A).

Treatment progress

**Figure 5:**
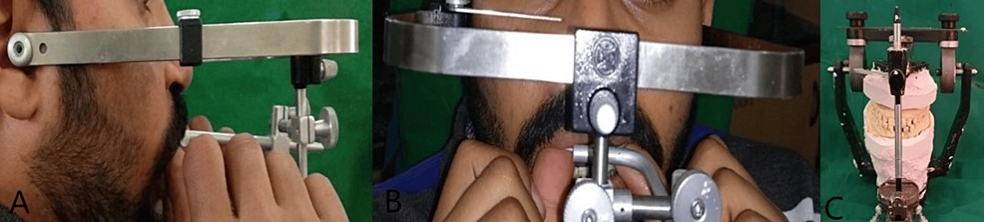
Pre-surgical facebow transfer (A) Profile. (B) Frontal. (C) Articulation of facebow transfer using Hanau’s articulator.

**Figure 6:**
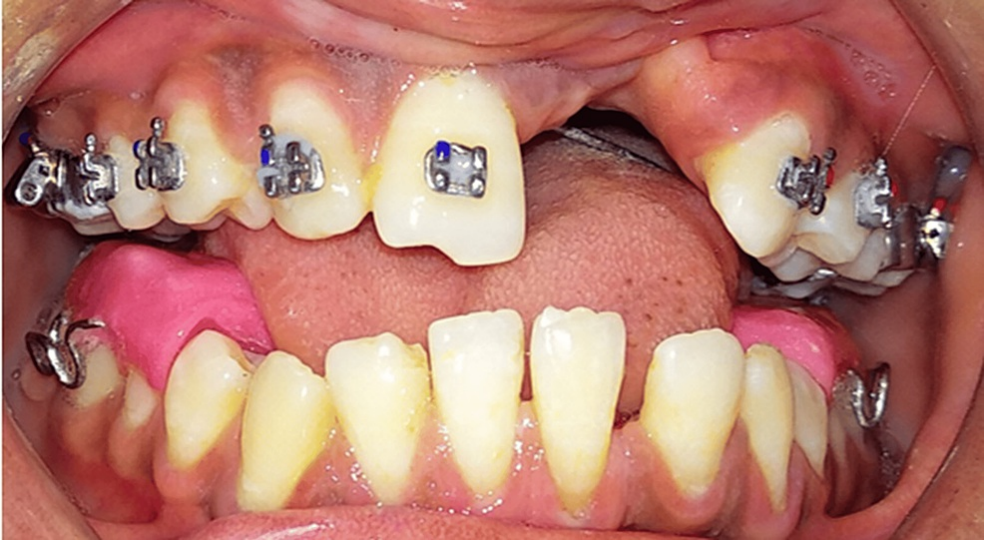
Removable posterior bite plane Frontal in occlusion view.

Initial leveling and alignment were performed on the maxillary arch using 0.022” slot MBT brackets. Alignment continued until 0.017” × 0.025” stainless steel archwire was achieved and was placed for one month. Subsequently, the patient was advised to undergo Le Fort I surgery for the correction of maxillary retrusion and closure of the maxillary space present due to a cleft on the left side. The decision for Le Fort I surgery was based on an evaluation of the lateral cephalogram, which showed a retropositioned maxilla. Post-alignment of the maxillary arch and facebow transfer was done to perform correction surgery on casts to acknowledge the outcome of surgery. The facebow used for this procedure was a spring bow (Hanau’s facebow) (Figure 5). The rationale for using a facebow is that it guarantees that the castings are positioned correctly with regard to the centers of lateral movements and the inter-condylar axis when mounted centrally. When it is essential to replicate the patient’s exact opening and closing motion with an articulator, it should be used. By using the orbitale as a third point of reference, it aligns the occlusal plane with the Frankfort horizontal plane. Pre-surgery, a crossbite was seen on the left side. To correct the crossbite, a removable posterior bite plane was fabricated to allow upper arch alignment (Figure 6).

**Figure 7:**
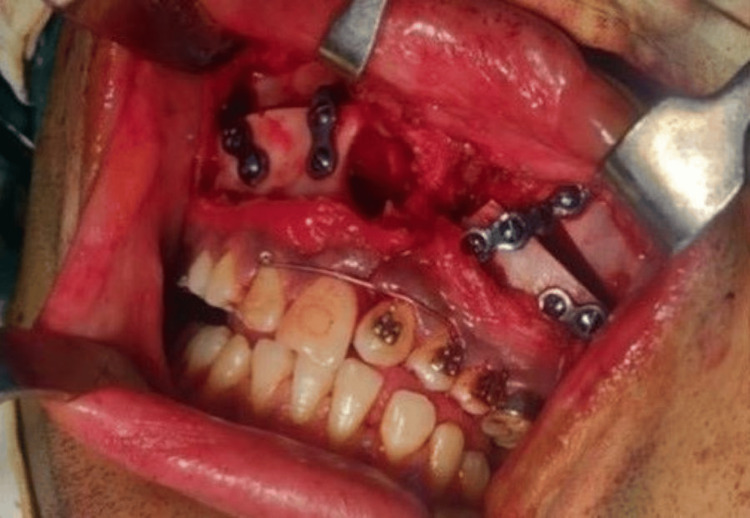
Le Fort I surgery Intra-oral photograph taken during surgery.

**Figure 8:**
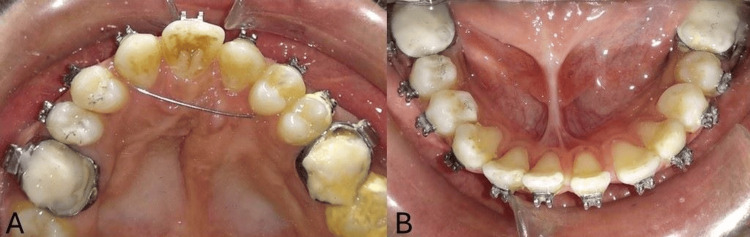
Post-surgical intra-oral photographs (A) Maxillary arch. (B) Mandibular arch.

**Figure 9:**
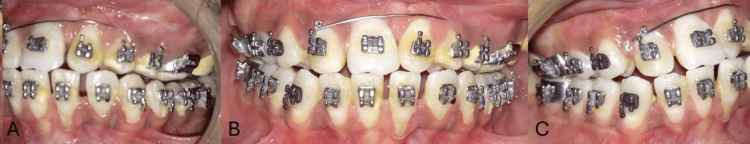
Post-surgical intra-oral radiograph (A) Right occlusal. (B) Frontal. (C) Left occlusal.

**Figure 10:**
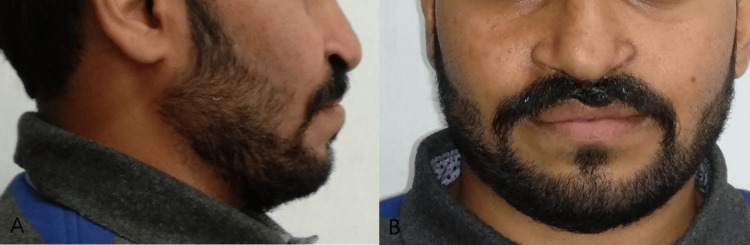
Post-surgical extra-oral photographs (A) Profile. (B) Frontal.

The goals of surgery were to achieve maxillary advancement, close the wide alveolar cleft, correct the skeletal malocclusion, and be aesthetic (correction of the facial profile). To skip the tedious procedure of ABG, it was suggested as an option to close the space by bringing the smaller segment toward the bigger segment, i.e., bringing the segment from the left side to the right side and closing the space completely, in addition to Le Fort I surgery by bringing both segments anteriorly (Figure 7). Post-advancement, the segments were stabilized using screws and arch bars, as seen in Figure 7. Once the segments were stabilized, the intra-arch wire was tied in the anterior segment, involving teeth from the right canine to the left first premolar, acting as a splint. Post-surgery, the patient was advised to visit after three months of recovery for follow-up and to evaluate further orthodontic treatment. During his first visit to the department after surgery, the mandibular arch was bonded to continue its alignment and closure of spaces in the anterior tooth region, and for correction of proclination, a multiloop archwire fabricated using 0.016 AJW archwire was placed. Radiographic records were taken to assess the corrections achieved through surgery using a lateral cephalogram, and both intra-oral and extra-oral photographs were taken (Figure 4B, Figure 8, Figure 9, and Figure 10). Following this, the patient decided to discontinue treatment at our hospital and shifted to another hospital as he relocated to a different city.

## Discussion

The presented case report focuses on the treatment of an adult patient with UCLP, offering insights into the challenges and successes encountered in managing this complex condition in adulthood [4].

Late presentation and challenges

The delayed presentation of UCLP in adulthood poses unique challenges compared to cases addressed during childhood. Adult patients often face not only aesthetic concerns but also functional issues, including speech difficulties, dental misalignment, and psychological impact. The case report underscores the importance of addressing both aesthetic and functional aspects to improve the overall quality of life for adult individuals with UCLP [5].

Comprehensive treatment planning

The treatment plan outlined in the case report likely involved a multidisciplinary team comprising plastic surgeons, orthodontists, oral and maxillofacial surgeons, and other specialists. A comprehensive evaluation would have been essential to understand the specific needs and challenges of the adult patient. Addressing dental and skeletal issues, as well as considering speech rehabilitation, likely played a significant role in the treatment planning process [6].

Surgical interventions

The case report may detail the surgical interventions performed to address the CLP in adulthood. Surgical techniques in adult UCLP cases often include lip revision surgeries, palate repairs, and ABG to enhance the functional and aesthetic aspects. The discussion may delve into the intricacies of these surgeries, the challenges encountered, and the outcomes achieved [7].

Orthodontic considerations

Orthodontic treatment is crucial in adult UCLP cases for dental alignment and occlusal correction. The discussion may elaborate on how orthodontic interventions were integrated into the treatment plan, addressing issues such as malocclusion, missing teeth, and other dental anomalies associated with UCLP [8].

Psychosocial impact and patient satisfaction

Adults with UCLP often carry the burden of long-standing aesthetic and functional challenges. The case report may discuss the psychosocial impact of the condition on the patient and how the treatment contributed to improved self-esteem and overall well-being. Patient satisfaction and subjective experiences should be considered and reported to provide a holistic view of the outcomes [9]. The stability of the treatment outcomes in adult UCLP cases is a critical consideration. The discussion should touch upon the long-term follow-up and stability of the achieved results. This includes any necessary orthodontic or surgical revisions over time and the maintenance of functional and aesthetic improvements [10].

## Conclusions

This case report contributes valuable insights into the challenges and successes of treating adult UCLP patients. It shows the need for a multidisciplinary approach to treating CLP cases for the best outcome. The discussion may suggest future directions for research and clinical practice, emphasizing the ongoing need for comprehensive and multidisciplinary approaches to address the unique aspects of UCLP in adulthood. Sharing such case reports contributes to collective knowledge and helps refine treatment strategies for adult individuals with CLP.
